# Correlation of Mechanical Thresholds, Glasgow Composite Measure Pain Scale, and Sharp and Wheeler Grading Scale in Dogs with Acute Thoracolumbar Disc Extrusions

**DOI:** 10.3390/ani15152176

**Published:** 2025-07-24

**Authors:** Jacqueline Hölscher, Alexandra Friederike Schütter, Sebastian Meller, Sabine B. R. Kästner, Holger Volk

**Affiliations:** 1 Department of Small Animal Medicine and Surgery, University of Veterinary Medicine Hannover, 30559 Hannover, Germany; jacqueline.hoelscher@tiho-hannover.de (J.H.); alexandra.schuetter@tiho-hannover.de (A.F.S.); sebastian.meller@tiho-hannover.de (S.M.); sabine.kaestner@tiho-hannover.de (S.B.R.K.); 2 Center for Systems Neuroscience Hannover, 20559 Hannover, Germany

**Keywords:** mechanical thresholds, Glasgow Composite Measure Pain Scale, Sharp and Wheeler Grading Scale, intervertebral disc extrusion, pain, dog

## Abstract

In dogs with intervertebral disc extrusion (IVDE), the Glasgow Composite Measure Pain Scale—Short Form (GCMPS) and the Sharp and Wheeler Grading Scale (SWGS) are routinely used in the evaluation of pain (GCMPS) and neurological function (SWGS). Additionally, quantitative sensory tests (QSTs) are increasingly being incorporated into veterinary clinical practice for pain characterisation. The aim was to investigate a possible relationship between the GCMPS, the SWGS, and mechanical thresholds (MTs) in 31 client-owned dogs with thoracolumbar IVDEs. Dogs were always assessed in the same order, starting with pain rating using the GCMPS, followed by classifying neurological severity using the SWGS, before determining MTs using a handheld pressure algometer. Dogs were evaluated over a five-day testing period (before surgery and on days one, two, three, and ten after surgery). The GCMPS and the SWGS data remained consistent across all days of testing. No statistically significant correlation or difference was observed between the scores. MTs showed a significant negative correlation with the GCMPS (r = −0.311; *p* < 0.001) and a positive one with the SWGS (r = 0.282; *p* = 0.002). The GCMPS and MTs showed a slight divergence in their progression. MTs might be more sensitive than GCMPS in reflecting clinical improvement and should be considered for clinical practice.

## Simple Summary

Intervertebral disc extrusions are one of the most common neurological disorders in dogs. The compression caused by the extruded disc material leads to neurological deficits such as paraparesis and pain. The objective of this study was to investigate and describe the relationship between neurological severity and pain in dogs with intervertebral disc extrusion. This was investigated using (1) the Glasgow Composite Measure Pain Scale—Short Form, (2) the Sharp and Wheeler Grading Scale, and (3) mechanical threshold testing. The results showed both scores (1) and (3) did not correlate with each other but also did not show any significant differences. In contrast, mechanical thresholds correlated with the neurological severity. Therefore, the measurement of mechanical thresholds could be an additional tool for assessing mechanical sensitivity in dogs with intervertebral disc extrusions.

## 1. Introduction

Thoracolumbar intervertebral disc extrusion (TL-IVDE) is a common spinal cord injury (SCI) in small chondrodystrophic dogs [1] with motor deficits and pain among the most common clinical signs of TL-IVDE [2]. Therefore, the examination of affected animals includes pain assessment and neurological severity grading [3]. Numerous severity grading scores, including pain evaluation, have been published for the assessment of neurological clinical signs and pain. However, the relatively subjective severity classification system developed by Sharp and Wheeler [4] is frequently employed in clinical practice due to its simple yet reliable prognostic scoring system. The scores range from normal (0) to plegic with loss of deep pain perception [5].

The Glasgow Composite Measure Pain Scale (GCMPS) is a somewhat subjective pain assessment tool validated for evaluation of acute post-operative pain in clinical patients [5,6]. It is a behaviour-based scale that assesses dogs in six categories: vocalisation, attention to wound, mobility, response, demeanour, and posture/activity. Points are assigned to each category (ranging from 0 to 4) and subsequently summed. If the calculated score exceeds 5 out of 20 points, an increase in analgesia is recommended [5,6,7,8]. Pain assessment via the GCMPS and the evaluation of neurological severity using the SWGS are well-established methods in clinical settings [4,5,8,9].

Quantitative sensory testing (QST) is a more objective possibility for pain assessment. By evaluating responses from the patient upon exposure to standardised stimuli, e.g., touch, pressure, or temperature, a somatosensory profile can be obtained, allowing identification of painful conditions or nerve damage [10,11,12]. One method used during QST is evaluation of mechanical thresholds (MTs) using a pressure algometer [13]. Mechanical algometers engage a complex array of sensory receptors and pain pathways and hence are rendered versatile tools for comprehensive pain assessment [13,14,15,16]. This type of investigation allows for the sensitisation of C-fibres and enables the assessment of mechanical nociceptive thresholds, the evaluation of pain associated with deeper tissue compression, and the investigation of pain phenomena such as mechanical hyperalgesia. The evaluation of sensory thresholds and pain characterisation through QST has been explored in veterinary research. Despite this progress, QST has yet to be established as a standardised clinical tool [10,12,17,18,19]. One approach that has been utilised in various veterinary studies is the use of pressure algometers, e.g., in cats [20], horses [21], and dogs [22,23].

To date, no studies have investigated the relationship between the GCMPS and SWGS, nor between GCMPS and MTs or SWGS and MTs in dogs. A study investigating possible benefits of pregabalin add-on analgesia in dogs after hemilaminectomy did measure pain-related behaviour via GCMPS and also evaluated mechanical pressure tolerance (MTs), but no statistical correlation between the two measures was performed [22]. However, in cats no correlation between scores of the Glasgow Feline Composite Measure Pain Scale and mechanical thresholds using a Small Animal ALGOmeter could be detected [24]. Given that both prognosis and healing duration are influenced by pain severity and the neurological status of the individual patient [25,26,27,28], it is of significant interest to explore whether a clinical association exists between these parameters in dogs.

Therefore, the aim of this study was to analyse these relationships while also incorporating and comparing the findings with another objective pain assessment, specifically MTs. The main hypothesis was that the GCMPS and SWGS are closely related, as well as the GCMPS and MTs. It was expected that the GCMPS would decrease with ongoing convalescence while MT might increase. Since extruded intervertebral disc material compresses the spinal cord, leading to both neurological deficits and pain [29,30,31], we further hypothesised that the GCMPS and the SWGS would show a similar trend in scores over a period of five days.

## 2. Materials and Methods

### 2.1. Animals

Thirty-one client-owned dogs were prospectively examined between November 2023 and August 2024 at the Department of Small Animal Medicine and Surgery, University of Veterinary Medicine Hannover. The owners were informed about the testing procedures and provided their written consent. The study was approved by the local ethics and animal welfare committee.

Dogs were included in this study if they weighed less than 20 kg and presented with clinical neurological signs suggestive of an IVDE. Analgesic treatment administered by the referring veterinarian or the emergency department did not hinder study inclusion.

Dogs were excluded from the study if the main investigator (JH) subjectively judged them to show aggressive behaviour (e.g., dogs that attempted to bite or were growling) or increased anxiety (e.g., dogs that were trembling, panting, or that attempted to escape). Assessment using a formal demeanour score was not performed. Furthermore, patients were not included in whom MRI scans (3.0 Tesla MRI SmartPath to dStream for XR; Philips Medical Systems GmbH, Hamburg, Germany) confirmed a diagnosis other than IVDE between the third thoracic and sixth lumbar vertebrae or if surgical treatment via hemilaminectomy was not necessary.

### 2.2. Data Collection

A complete initial general and neurological examination was performed on each dog by a neurological specialist or specialist in training (ECVN resident or ECVN or ACVIM diplomate). Follow-up examinations were always performed in the same order by the same investigator (JH). They included a general and neurological examination, as well as pain evaluation using GCMPS, followed by neurological severity assessment using SWGS and lastly QST by assessing MTs. A German version of the GCMPS—Short Form was utilised. Since all enrolled dogs were paraparetic or paraplegic, the mobility assessment portion of the GCMPS was omitted [22]. The GCMPS ranges from 0 to 20, while the SWGS ranges from 0 to 5.

All assessments were conducted over a ten-day study period, with a total of five testing time points (pre-surgery and days one, two, three, and ten after surgery). Furthermore, the dogs would have been receiving pain medication throughout their hospital stays at the discretion of the attending clinician. In some cases, animals that had been discharged earlier returned for follow-up testing. Evaluation of MTs using pressure algometry as one method of quantitative sensory testing was chosen for the comparisons during this trial due to its simplicity, intuitiveness, and high analytical capabilities. MTs were determined using a handheld pressure algometer (TopCat Metrology, Ely, UK) with a round, 2 mm diameter pin. It was placed vertically on the selected, clipped skin area with even pressure. MT measurements were taken three times in a randomised order at each of the eight different test sites. These were located bilaterally two to four centimetres lateral to the dorsal spinal processes, depending on the size of the dog, at the levels of Th11, Th13, L2, and L4. Pressure was manually applied using a constant rate of 2 N/s until the dog showed a purposeful reaction. This could be a clear avoidance behaviour, such as head turning or withdrawal from the stimulus or vocalisation. Once the end point was reached, the measurement was stopped, and the respective value recorded. For evaluation of the dog’s reaction, a second veterinary professional (vet, veterinary nurse, or final-year vet student) was present. Analgesic therapy was adjusted according to GCMPS.

### 2.3. Statistical Analysis

Demographic data of the patients, including breed, sex, and age, were analysed. MT values from all test sites per dog, the GCMPS scores, and the SWGS scores were calculated as median values for each testing timepoint and visualised using GraphPad Prism (GraphPad Prism 10.4.1, GraphPad Software, Inc., La Jolla, CA, USA). The relationships between MTs, GCMPS, and SWGS were assessed using Spearman rank correlation analyses. To detect differences within each system among individual test days across the entire testing period, mixed-effects analyses were performed, with timepoints representing the fixed effect, followed by Tukey’s multiple comparisons tests. A Wilcoxon matched-pairs signed rank test was performed to determine the significance between SWGS and GCMPS across the entire testing period. All tests were two-sided, and a *p*-value less than 0.05 was considered significant [32].

## 3. Results

Thirty-one dogs were included (six female (19%), five female-neutered (16%), 12 male (39%), and eight male-neutered dogs (26%)). The median age was 5.42 years (range 2.17–12.83), and the median body weight was 10.2 kg (range 3.8–18.5). The breeds represented were eleven (35%) French Bulldogs, six (19%) Dachshunds, eight (26%) mixed-breed dogs, and six (20%) other dog breeds (Poodle, Croatian Shepherd Dog, Chihuahua, Boloka Zwetna, Cavalier King Charles Spaniel, and Shih Tzu).

**Figure 1:**
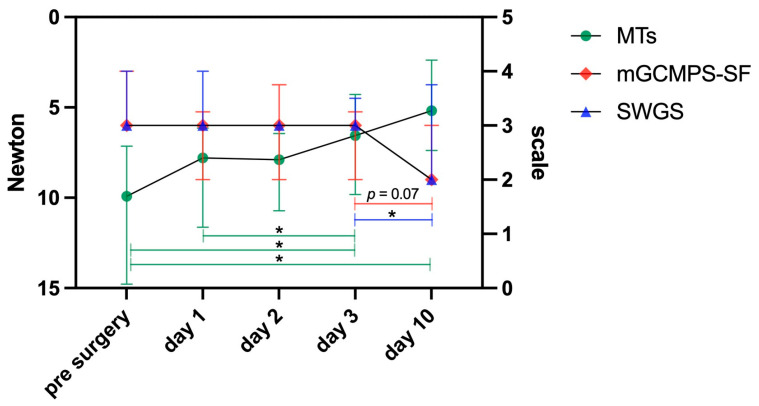
Data points represent medians with interquartile ranges of the mechanical thresholds (green dots) in Newton (relative to the left *y*-axis) and of scores from the modified Glasgow Composite Measure Pain Scale—Short Form (GCMPS; red diamond symbols) as well as the Sharp and Wheeler Grading Scale (SWGS; blue triangles), both in relation to the right *y*-axis. The right *y*-axis displays values between 0 and 5. Theoretically, GCMPS scores can reach 20, but as scores in the current study did not exceed 4, it was decided for the current *y*-axis to allow better visualisation in comparison to SWGS scores. Data of 31 dogs with acute, painful thoracolumbar intervertebral disc extrusions. Please note that the left *y*-axis displays the values in the opposite direction than might be expected, in contrast to the right *y*-axis. This is meant to better illustrate that values for all three systems in the lower part of the graph are more likely to correspond to less sensitive animals, while in the upper part, they indicate more sensitive animals. Higher data of GCMPS correspond to animals in more pain, and higher scores of SWGS to higher neurological severity of the animals. Mixed-effects models with timepoints of measurements as the fixed effect were used for each system separately, followed by Tukey’s multiple comparisons tests in order to conduct inter-day comparisons (* *p* < 0.05). Only the significant comparison between day 3 and day 10 for the SWGS is shown representatively, whereas all comparisons with day 10 were significant for SWGS (*p* < 0.05).

For the evaluation of data from GCMPS, SWGS, and MTs, medians were calculated for all dogs per test and day. No statistical differences between medians of GCMPS and SWGS of all dogs were detectable over the entire study period (*p* = 0.125). Severity of neurological impairment, assessed via SWGS, differed on all days significantly from day 10 (mixed-effects model, *p* = 0.011, Tukey’s multiple comparisons with *p* < 0.05). The GCMPS scores showed a trend to decrease between day 3 and day 10 (*p* = 0.07). MTs decreased significantly over time (*p* < 0.01), indicating increased sensitivity, with significant differences between pre-surgery and day 3 (*p* = 0.029) and day 10 (*p* = 0.013), as well as between day 1 and day 10 (*p* = 0.029) (Figure 1). MT values and GCMPS scores from all dogs and days were weakly negatively correlated (i.e., GCMPS decreased, and MT decreased; r = −0.311, *p* < 0.001). MT values and SWGS scores exhibited a weak positive correlation (r = 0.282, *p* = 0.002). No correlation was found between GCMPS and SWGS (r = 0.139; *p* = 0.133).

### Pain Medication

Analgesic treatment of all study patients was based on an initially standardised analgesic protocol (Table 1). Analgesic adjustments were made based on a clinically routinely performed 4-hourly GCMPS pain assessment. In case a dog appeared to be in pain (scored > 5), additional analgesia was provided to secure animal wellbeing. To achieve this, either, if possible, the fentanyl–lidocaine–ketamine continuous drip infusion (FLK-CDI) rate was adjusted back to the initial dose displayed in Table 1, or additional methadone at a dose of 0.15–0.3 mg/kg was given intravenously (IV). In dogs scoring ≤ 3 in 2 assessments in a row, dosing of analgesics was decreased to prevent overdosing and possibly concurrent side effects.

## 4. Discussion

This study aimed to investigate the relationship between the GCMPS and SWGS, along with incorporating MT testing results.

GCMPS and SWGS did not significantly correlate or differ across time points, which may be intrinsically linked to the pathogenesis of IVDE. The interaction between pain and extent of neurological deficits can be underscored when considering that the prognosis and healing time for patients with TL-IVDE depend on both neurological severity [28] and pain intensity [25,26,27]. Compression of the meninges and nerve roots by extruded disc material results in neurological deficits and pain, initiating an inflammatory process [29,30,31]. In related human studies, concentrations of inflammatory mediators measured 72 h after spinal cord injury were directly linked to the injury’s severity [33,34]. Expecting that increased inflammation would lead to increased pain, a connection between magnitude of pain and severity of neurological injury could have been anticipated.

The observation that GCMPS and SWGS scores remained consistent from the pre-MRI period to day three may be associated with ongoing inflammation, and therefore it is also likely that pain persists, despite surgical decompression offering pain relief [35], for days or weeks [36] without guaranteeing immediate pain relief [37]. This post-injury inflammation, along with eventual necrosis and apoptosis among neurons and oligodendrocytes and scar tissue formation, further delays spinal cord functional recovery, supporting the notion that pain and neurological severity are interconnected [33,34,38]. The GCMPS remaining consistent could likely also be attributed to the adjustment of analgesic treatment according to the GCMPS. Individual analgesic therapy was adjusted for each animal following assessment with the GCMPS, as this is clinically indicated and minimises the risk of both under- and over-dosing. However, this individualised approach may limit the validity of longitudinal analyses based on GCMPS scores. From a scientific perspective, uniform and consistent analgesic administration across all subjects would have been methodologically preferable. Nevertheless, such standardisation was not ethically justifiable in the context of this clinical study, where individualised treatment was essential. As a result, the findings of this investigation are directly translatable to clinical practice, providing a sound basis for evidence-based clinical decision-making under real-world conditions. The additional assessment of MTs on the test days influenced the adjustment of analgesic treatment only to a limited extent.

The lack of correlation between GCMPS and SWGS may be attributed, in part, to the distinct measurement focuses of the two scales. The GCMPS is a validated instrument for the assessment of acute postoperative pain, based primarily on the observation of specific behavioural responses. These responses are classified into defined categories, thereby facilitating the clinical decision-making process regarding the need for adjustment of analgesic therapy [5,6,7]. Additionally, in this study, due to the presence of paraparesis or paraplegia, the ‘mobility’ category of the GCMPS was not assessed. In contrast, the SWGS is a clinical scoring system designed to assess the severity of neurological dysfunction. Originally developed for thoracolumbar intervertebral disc disease, it evaluates residual motor function through systematic gait analysis [4]. Although both instruments assess distinct clinical dimensions (nociception versus neurological function), the underlying pathophysiology of IVDE often involves both components. Consequently, a correlation between pain severity and neurological impairment could have been anticipated; however, such an association was not demonstrated in the present study.

Notably, we compared GCMPS with MTs to broaden pain assessment insights. The GCMPS was selected due to its extensive validation and longstanding clinical use [6,7,39]. MTs were evaluated, as QST serves as a recognised objective pain assessment method in human medicine, establishing its role in veterinary practice [12,22,23,40]. However, MTs and GCMPS scores were weakly negatively correlated, and SWGS scores exhibited a significant weak positive correlation with MTs. This suggests that a more objective measure of pain, such as MTs, could be more sensitive to improvement in clinical status, correlating deep pressure sensation assessment with improved neurological function. Interestingly, in contrast to GCMPS, MT levels did significantly, albeit weakly, correlate with improvement in neurological status. This could suggest that MTs may be a more sensitive assessment tool than GCMPS. In general, pressure algometry has been shown to be reliable in identifying areas of induced pain and hyperalgesia, as well as regions of hypoalgesia, reinforcing its clinical utility in pain assessment [21,41]. It is also possible that using two pain assessment systems may capture different types of pain, leading to these divergent results. Discrepancies may also arise because GCMPS relies on an ordinal-based scale, whereas MTs offer more precise numerical data [6]. When interpreting the significant increases in MT data, potential influences of hyperalgesia [15,42] and learnt aversion due to repeated measurements must be considered [43]. MT is only one method included in human static QST; incorporating additional or dynamic methods, such as assessment of conditioning pain modulation, might have given a more comprehensive picture of the somatosensoric system of the animals. Given the lack of canine reference values, as is the case with many objective methods routinely used in human medicine, pain assessment in dogs remains difficult—particularly because only behavioural responses can be interpreted [44]. Nonetheless, pain levels might be inferred from the condition’s neurological severity.

Between day three and day ten, a significant improvement in the SWGS was observed, while a trend towards improvement was noted in the GCMPS evaluation. Concurrently, the mechanical thresholds indicated an increased sensitivity. With regard to the increased sensitivity to the pressure stimulus, one consideration could be that MT measurements capture deep pain sensation, which could be reduced in the surrounding tissue after an IVDE, and that the increase in sensitivity is a return to normal. The increased sensitivity could be related to progressive tissue healing, which may have contributed to the restoration of previous physiological responses. The correlation of MT to SWGS over the course of the testing days and the results on day ten with GCMPS suggesting less pain and MT showing an increase in sensitivity could support this hypothesis. However, the extent to which this played a role is unclear and should be investigated in future studies and also be considered when used in the clinical settings. A further potential explanation could also be the development of primary hyperalgesia, which might be more important for IVDE and post-surgical pain assessment. C-fibres, which are stimulated by the pressure algometer, can be sensitised by tissue damage and lead to peripheral sensitisation and primary hyperalgesia [15,42,45,46]. The GCMPS primarily relies on behavioural assessments, where only one criterion involves light palpation of potentially painful areas [5,6]. These findings suggest that the improvement in the GCMPS score may not be solely attributed to a reduction in pain intensity in the lumbar region. Rather, the high proportion of behavioural assessment in the GCMPS calculation may play a role. As some animals tested on day 10 had already been discharged to home care prior to the test, the familiar environment and contact with familiar caregivers may have positively influenced the behaviour and overall emotional state of the animals. In comparison, MTs evaluations could be more specific for pain induced by pressure of deeper tissue areas, as the direct signalling pathway is probably less influenced by emotions. However, as only MTs were used for comparison in this study, a complete somatosensory profile of the patients is not available. This should be taken into account when interpreting the contrasting trends observed between GCMPS and MTs. A comprehensive comparison of pain assessed through full QST modalities and the GCMPS might yield different results.

The study’s limitations include the small sample size of dogs tested. Further, only one observer who was aware of the diagnosis and treatment assessed the animal. Due to the clinical nature of the study and to minimise discomfort to the dogs, it was decided against including multiple observers. In a future study, a setting including multiple observers to enable calculation of inter-observer agreement would be interesting. This could also mitigate subjectivity in GCMPS evaluation. In such a setting a more comprehensive dataset could be gained, and possibly more reliable reference values for MTs could be established. Further ongoing research should encompass dogs with other spinal cord injuries to validate and strengthen the current findings. A standardised assessment time should also be established to control potential analgesic influences. Furthermore, the analgesic treatment may have influenced the current study and the analysis of the GCMPS, as every patient’s pain treatment was individually adapted according to its GCMPS score to ensure animal wellbeing. Another limitation of this study is that, to date, only mechanical threshold values have been correlated with the GCMPS and SWGS, thereby representing only a partial assessment toward a comprehensive somatosensory profile of the patients. Quantitative sensory testing (QST) includes not only the evaluation of mechanical sensitivities but also, among other modalities, the assessment of tactile sensitivity (e.g., using von Frey filaments) and sensitivity to temperature changes (e.g., via cold stimulation) [10,11,12]. However, since mechanical threshold determination was demonstrated in a previous study to be simple, intuitive, and suitable for clinical use in dogs [23,47,48], only MTs were included in the context of the present comparison. In addition, the correlations between MTs and the SWGS, as well as between MTs and the GCMPS, yielded relatively low r-values, indicating statistically weak associations. Given the fundamentally different scoring systems and measurement constructs used, this finding is not unexpected, but it should nonetheless be acknowledged. At present the clinical relevance of this statistical finding is not clear and should be investigated in a larger population.

## 5. Conclusions

In dogs with acute, painful thoracolumbar IVDE, postoperative improvement of neurological status evaluated via SWGS weakly correlated with MT measurements, but not GCMPS scores. Therefore, in future, mechanical sensitivity testing could possibly be used as an additional tool to monitor neurological improvement in a more objective way.

## Figures and Tables

**Table 1:** Overview of the analgesics used and their respective dosage regimens. Note: In two patients, pregabalin was replaced by carprofen, and only one patient received gabapentin. The administration of methadone and adjustment of the fentanyl–lidocaine–ketamine continuous drip infusion (FLK-CDI) were dependent on the individual pain score. Quarter in die (QID), intravenously (IV), ter in die (TID), peroral (PO), and semel in die (SID).

Analgesic	Dosage
Metamizole (Novalgin, Sanacorp, Planegg, Germany)	35–50 mg/kg QID (IV) D10 35–50 mg/kg TID
Pregabalin (PregaTab^®^, Sanacorp)	2–4 mg/kg TID (PO)
Carprofen (Rimadyl^®^, Zoetis Deutschland GmbH, Berlin, Germany)	4 mg/kg SID (PO; only in two patients)
Methadone (Comfortan^®^, Dechra Limited, Aulendorf, Germany)	0.15–0.3 mg/kg (IV; single dose up to three times daily)
Gabapentin (Gabapentin, Sanacorp) Fentanyl (Sanacorp) Lidocaine (B. Braun Vet Care GmbH, Melsungen, Germany) Ketamine (CP-Pharma GmbH, Burgdorf, Germany) CDI	5 mg/kg TID (PO; only in one patient) 0.001 mg/kg/h fentanyl (IV) 0.6 mg/kg/h lidocaine (IV) 0.12 mg/kg/h ketamine (IV)

## Data Availability

The original contributions presented in the study are included in the article; further enquiries can be directed to the corresponding author/s.

## Abbreviations

The following abbreviations are used in this manuscript:

FLK-CDI	Fentanyl–lidocaine–ketamine continuous drip infusion
GCMPS	Glasgow Composite Measure Pain Scale
IVDE	Intervertebral disc extrusion
L	Lumbar vertebra
QID	Quarter in die
SCI	Spinal cord injury
SID	Semel in die
SWGS	Sharp and Wheeler Grading Scale
Th	Thoracic vertebra
TID	Ter in die
TL-IVDE	Thoracolumbar intervertebral disc extrusion
